# The transcription factor IbNAC29 positively regulates the carotenoid accumulation in sweet potato

**DOI:** 10.1093/hr/uhad010

**Published:** 2023-02-01

**Authors:** Shihan Xing, Ruijie Li, Haoqiang Zhao, Hong Zhai, Shaozhen He, Huan Zhang, Yuanyuan Zhou, Ning Zhao, Shaopei Gao, Qingchang Liu

**Affiliations:** Key Laboratory of Sweet Potato Biology and Biotechnology, Ministry of Agriculture and Rural Affairs/Beijing Key Laboratory of Crop Genetic Improvement/Laboratory of Crop Heterosis and Utilization, Ministry of Education, College of Agronomy & Biotechnology, China Agricultural University, Beijing 100193, China; Key Laboratory of Sweet Potato Biology and Biotechnology, Ministry of Agriculture and Rural Affairs/Beijing Key Laboratory of Crop Genetic Improvement/Laboratory of Crop Heterosis and Utilization, Ministry of Education, College of Agronomy & Biotechnology, China Agricultural University, Beijing 100193, China; Key Laboratory of Sweet Potato Biology and Biotechnology, Ministry of Agriculture and Rural Affairs/Beijing Key Laboratory of Crop Genetic Improvement/Laboratory of Crop Heterosis and Utilization, Ministry of Education, College of Agronomy & Biotechnology, China Agricultural University, Beijing 100193, China; Key Laboratory of Sweet Potato Biology and Biotechnology, Ministry of Agriculture and Rural Affairs/Beijing Key Laboratory of Crop Genetic Improvement/Laboratory of Crop Heterosis and Utilization, Ministry of Education, College of Agronomy & Biotechnology, China Agricultural University, Beijing 100193, China; Key Laboratory of Sweet Potato Biology and Biotechnology, Ministry of Agriculture and Rural Affairs/Beijing Key Laboratory of Crop Genetic Improvement/Laboratory of Crop Heterosis and Utilization, Ministry of Education, College of Agronomy & Biotechnology, China Agricultural University, Beijing 100193, China; Key Laboratory of Sweet Potato Biology and Biotechnology, Ministry of Agriculture and Rural Affairs/Beijing Key Laboratory of Crop Genetic Improvement/Laboratory of Crop Heterosis and Utilization, Ministry of Education, College of Agronomy & Biotechnology, China Agricultural University, Beijing 100193, China; Key Laboratory of Sweet Potato Biology and Biotechnology, Ministry of Agriculture and Rural Affairs/Beijing Key Laboratory of Crop Genetic Improvement/Laboratory of Crop Heterosis and Utilization, Ministry of Education, College of Agronomy & Biotechnology, China Agricultural University, Beijing 100193, China; Key Laboratory of Sweet Potato Biology and Biotechnology, Ministry of Agriculture and Rural Affairs/Beijing Key Laboratory of Crop Genetic Improvement/Laboratory of Crop Heterosis and Utilization, Ministry of Education, College of Agronomy & Biotechnology, China Agricultural University, Beijing 100193, China; Key Laboratory of Sweet Potato Biology and Biotechnology, Ministry of Agriculture and Rural Affairs/Beijing Key Laboratory of Crop Genetic Improvement/Laboratory of Crop Heterosis and Utilization, Ministry of Education, College of Agronomy & Biotechnology, China Agricultural University, Beijing 100193, China; Key Laboratory of Sweet Potato Biology and Biotechnology, Ministry of Agriculture and Rural Affairs/Beijing Key Laboratory of Crop Genetic Improvement/Laboratory of Crop Heterosis and Utilization, Ministry of Education, College of Agronomy & Biotechnology, China Agricultural University, Beijing 100193, China

## Abstract

Carotenoid is a tetraterpene pigment beneficial for human health. Although the carotenoid biosynthesis pathway has been extensively studied in plants, relatively little is known about their regulation in sweet potato. Previously, we conducted the transcriptome database of differentially expressed genes between the sweet potato (*Ipomoea batatas*) cultivar ‘Weiduoli’ and its high-carotenoid mutant ‘HVB-3’. In this study, we selected one of these candidate genes, *IbNAC29*, for subsequent analyses. IbNAC29 belongs to the plant-specific NAC (NAM, ATAF1/2, and CUC2) transcription factor family. Relative *IbNAC29* mRNA level in the HVB-3 storage roots was ~1.71-fold higher than Weiduoli. Additional experiments showed that the contents of α-carotene, lutein, β-carotene, zeaxanthin, and capsanthin are obviously increased in the storage roots of transgenic sweet potato plants overexpressing *IbNAC29*. Moreover, the levels of carotenoid biosynthesis genes in transgenic plants were also up-regulated. Nevertheless, yeast one-hybrid assays indicated that IbNAC29 could not directly bind to the promoters of these carotenoid biosynthesis genes. Furthermore, the level of *IbSGR1* was down-regulated, whose homologous genes in tomato can negatively regulate carotene accumulation. Yeast three-hybrid analysis revealed that the IbNAC29-IbMYB1R1-IbAITR5 could form a regulatory module. Yeast one-hybrid, electrophoretic mobility shift assay, quantitative PCR analysis of chromatin immunoprecipitation and dual-luciferase reporter assay showed that IbAITR5 directly binds to and inhibits the promoter activity of *IbSGR1*, up-regulating carotenoid biosynthesis gene *IbPSY*. Taken together, *IbNAC29* is a potential candidate gene for the genetic improvement of nutritive value in sweet potato.

## Introduction

Carotenoids are pigments, widely distributed in nature, and are divided into two groups: (i) carotenes including lycopene and α/β/γ-carotene, and (ii) xanthophyll like lutein, zeaxanthin, and violaxanthin [1]. Over 750 natural carotenoids have been found in plants, algae, fungi, and bacteria [2, 3]. Interestingly, carotenoids not only are crucial in these organisms that can synthesize them, but also in animals and humans. Humans must obtain carotenoids in their diet because their body cannot synthesize them [4, 5].

In plants, carotenoids are biosynthesized via isopentenyl pyrophosphate (IPP) produced from the methylerythritol phosphate (MEP) pathway [6]. Phytoene synthase (PSY) is considered to be a major rate-limiting enzyme of carotenoid biosynthesis pathway. The subsequent cyclization of all-trans-lycopene by lycopene ε-cyclase (LCYE) and/or lycopene β-cyclase (LCYB) leads to the formation of symmetric orange β- and α-carotene in the β-β and β-ε branch, respectively. Then, ε-carotene hydroxylase (ECH) and β-carotene hydroxylase (BCH) add hydroxyl moieties to the cyclic end groups to produce lutein from α-carotene and zeaxanthin from β-carotene [7–9]. The epoxidation of zeaxanthin then produces antheraxanthin and violaxanthin [10], which are further converted by capsanthin-capsorubin synthase (CCS) into capsanthin and capsorubin, respectively [11, 12]. Although the key enzymes involved in the carotenoid biosynthetic pathway have been extensively studied, the mechanism regulating carotenoid biosynthesis is still not well-explained.

The plant NAC (NAM, ATAF1/2, and CUC2) protein family is involved in diverse biological processes, including lateral root formation, secondary cell wall synthesis, and vegetative organ and fruit development [13, 14]. Overexpression of *SlNAC1* decreases the levels of β-carotene, lycopene, and total carotenoid, while increasing the lutein content in tomato (*Solanum lycopersicum*) [14]. In *SlNAC4* RNA interference transgenic fruits, the total carotenoid level is significantly reduced after the break (B) stage [13, 15]. Similarly, in the B + 3 and B + 10 stages of the NAC transcription factor *SlNAC3* mutant, *nor-like1*, carotenoid levels are also significantly decreased [16]. On the contrary, overexpression of *SlNAC-NOR* significantly accelerates the fruit ripening process and produces higher carotenoid levels [17].

Besides, the MYB transcription factors also are important in regulating carotenoid biosynthesis. Based on the number of MYB conserved domains, MYBs are divided into (i) R1- (including one MYB domain); (ii) R2R3- (including two MYB domains); and (iii) R1R2R3-type MYB (including three MYB domains) subgroups. At present, mostly R2R3-type MYBs are reported to regulate carotenoid biosynthesis. For example, overexpressing *AdMYB7* causes the accumulation of carotenoids and chlorophyll in kiwifruit [18]. Conversely, downregulating the R2R3-MYB transcription factor *RCP1* (*Reduced carotenoid pigmentation 1*) expression reduces carotenoid content in *Mimulus lewisii* flowers [19]. Overexpression of *CrMYB68* (*Citrus reticulate*) negatively regulates the expression of *NbBCH2* and *NbNCED5* to suppress the transformation of α- and β-branch carotenoids in tobacco leaves [20]. Moreover, MYB transcription factors form complexes with other proteins to participate in pigment biosynthesis. In *Medicago truncatula*, the MtWP1-MtTT8-MtWD40-1 complex regulates flower pigmentation via the anthocyanin and carotenoid biosynthesis [21].

According to previous studies, STAY-GREEN (SGR) is an evolutionarily conserved chloroplast-targeted protein in higher plants which works in carotenoids biosynthesis, chlorophyll degradation and senescence [22, 23]. Silencing the *LeSGR1* (*Lycopersicon esculentum*) expression inhibits chlorophyll degradation in the leaves and fruits of tomato. Interestingly, SlSGR1 regulates lycopene and β-carotene accumulation by interacting directly with SlPSY1, a key carotenoid biosynthesis enzyme gene [24].

Sweet potato (*Ipomoea batatas* (L.) Lam. [2n = B_1_B_1_B_2_B_2_B_2_B_2_ = 6x = 90]) provides carbohydrates and carotenoids for humans and is one of the most important food crops across the world. Sweet potato, especially the orange-fleshed cultivars, contains high levels of β-carotene, which could combat vitamin A deficiency [25, 26]. In this study, we found that overexpression of *IbNAC29* significantly increases the carotenoid content. We also demonstrated that IbNAC29 participates in the carotenoid biosynthesis by forming a regulatory module with IbMYB1R1 (R1-type MYB) and IbAITR5. Moreover, IbAITR5 represses the transcription of *IbSGR1*. Our results further indicated that IbNAC29 might enhance this repression, thus resulting in the carotenoid accumulation.

## Results

### *IbNAC29* is a potential candidate gene for regulating the carotenoid biosynthesis pathway

**Figure 1:**
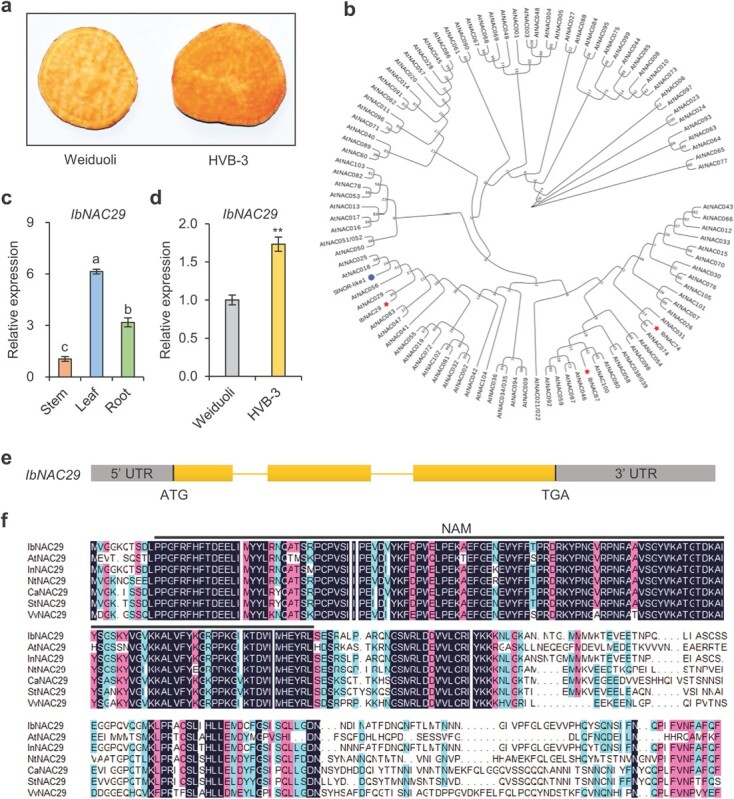
Molecular characterization of IbNAC29. **a** Phenotype of orange-fleshed sweet potato cultivar Weiduoli and its mutant HVB-3 with high carotenoid content. **b** Phylogenetic analysis of the NAC protein in *Arabidopsis* and sweet potato (IbNAC29, IbNAC74, IbNAC87) was performed with 1000 bootstrap iterations using the neighbor-joining method in MEGA 7.0. The numbers on the tree nodes represent 1000 repeated boot values. IbNAC29, IbNAC74, and IbNAC87 from carotenoid-related transcriptome data are marked with red stars. SlNOR-like1, a reported *NAC* transcription factor linked to carotenoid biosynthesis in tomato, is marked with a blue circle. **c** Relative mRNA level of *IbNAC29* in different tissues of 4-week-old *in vitro*-grown HVB-3 plants. *IbActin* was used as the internal control. **d** Relative mRNA level of *IbNAC29* in the storage roots of Weiduoli and HVB-3 at storage root expansion stage. *IbActin* was used as the internal control. Error bars indicate SD (*n* = 3). ^**^P < 0.01, respectively, by Student’s *t*-test. **e** Gene structure analyses of *IbNAC29*. Grey boxes indicate the untranslated region, including 5′ untranslated regions (UTRs) and 3′ UTR. Yellow boxes and lines represent exons and introns, respectively. **f** Multiple sequence alignment of NAC29 from different species. Plant species include *Arabidopsis thaliana* (At), *Ipomoea nil* (In), *Nicotiana tabacum* (Nt), *Capsicum annuum* (Ca), *Solanum tuberosum* (St), and *Vitis vinifera* (Vv). The NAM domain is represented by black lines.

Previously, we performed RNA sequencing analyses on sweet potato cultivar Weiduoli and its high-carotenoid mutant HVB-3 (Fig. 1a) to identify the differentially expressed genes [27]. Among these genes, the expressions of three NAC transcription factor genes, including *IbNAC29*, *IbNAC74*, and *IbNAC87*, were upregulated in HVB-3 [27]. As shown in Fig. 1b, *IbNAC29* is homologous to the *NAC* transcription factor *SlNOR-like1*. Regulation of carotenoid biosynthesis by *SlNOR-like1* in tomato has been reported recently [16]. Furthermore, *IbNAC29* was widely expressed in the leaf, stem, and root tissues of HVB-3 (Fig. 1c). Quantitative real-time PCR (qRT-PCR) analysis showed mRNA level in the storage roots of HVB-3 was ~1.71-fold higher than Weiduoli, thereby showing its potential link with carotenoid biosynthesis (Fig. 1d). Therefore, we selected *IbNAC29* for subsequent analyses.

The coding sequence of *IbNAC29* was 849 bp and contained three exons and two introns, encoding a protein of 282 amino acids (Fig. 1e). Based on the NCBI’s Conserved Domains Database [28], N-terminal region of IbNAC29 contains a highly conserved NAM DNA-binding domain (Fig. 1f).

### IbNAC29 is nuclear-localized and can function in transcriptional activation

**Figure 2:**
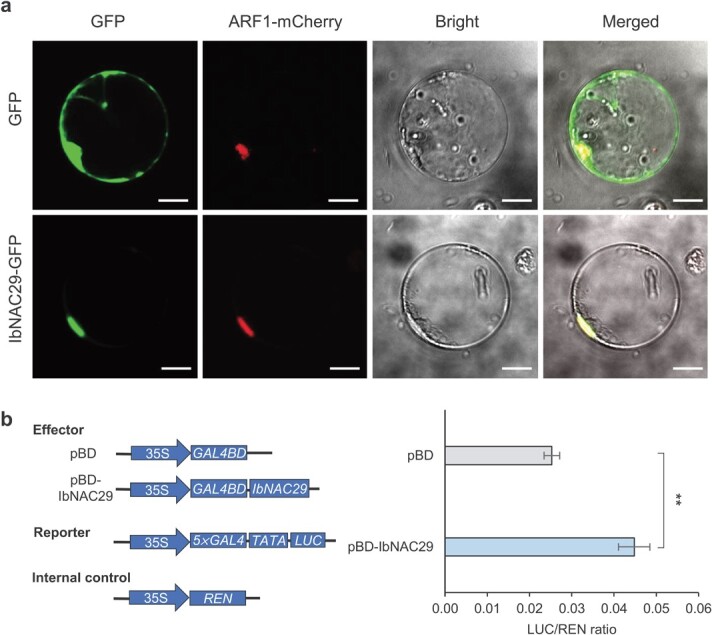
Subcellular localization and transcriptional activity of IbNAC29. **a** Subcellular localization of IbNAC29 in protoplasts. IbNAC29-GFP was co-transformed with ARF1-mCherry, which was used as a nuclear marker. Bar = 10 μm. **b** Transactivation assay of IbNAC29 in protoplasts. The GAL4 BD empty vector was used as a negative control. The expression level of REN was used as an internal control. Error bars indicate SD (*n* = 3). ^**^ indicates a significant difference from that of pBD at *P* < 0.01, by Student’s *t*-test.

To further study the subcellular localization of IbNAC29, we expressed the IbNAC29-GFP fusion protein in protoplasts. As a control, empty GFP plasmid was transfected into protoplasts. As shown in Fig. 2, GFP itself was distributed in the nucleus and the cytoplasm as expected, whereas the fusion protein IbNAC29-GFP was nuclear-localized (Fig. 2a). Furthermore, the position of green fluorescence from the IbNAC29-GFP fusion protein merged with the red fluorescence from the nuclear marker ARF1-mCherry [29], suggesting that IbNAC29 localizes to the nucleus.

Next, we used the transient expression system to investigate whether IbNAC29 acts as a transcriptional activator. We co-expressed the effector and reporter vectors in protoplasts, and quantified the luciferase activity after 16 h incubation. The results showed that the luciferase activity is significantly increased when IbNAC29 is co-expressed (Fig. 2b), thus indicating that IbNAC29 is a transcriptional activator.

### Overexpression of *IbNAC29* enhances carotenoid levels in the storage roots of sweet potato

**Figure 3:**
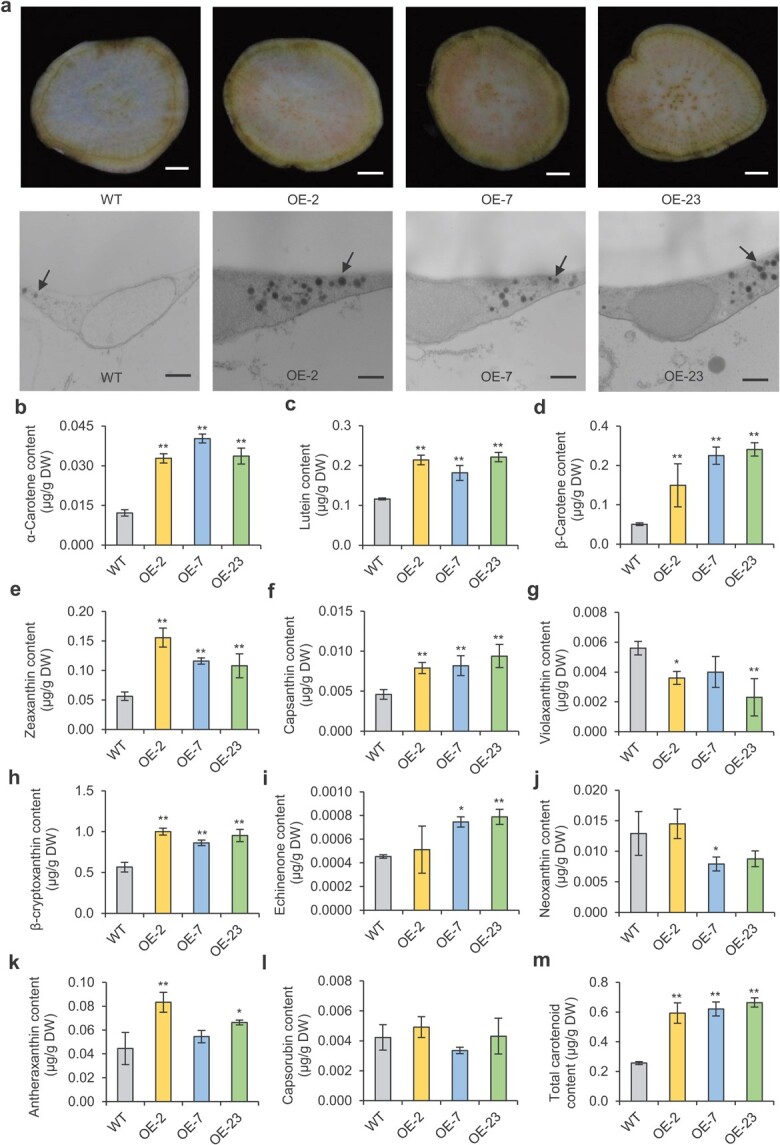
Overexpression of *IbNAC29* increases the carotenoid content in the storage roots of sweet potato during the maturity stages. **a** Storage roots’ transverse sections (up), Bar = 1 cm. The carotenoid globules (dark grey) are shown in the electron microscopy images (down), Bar = 500 nm. Arrows indicate the carotenoid globules. **B–l** Levels of α-carotene, lutein, β-carotene, zeaxanthin, capsanthin, violaxanthin, β-cryptoxanthin, echinenone, neoxanthin, antheraxanthin, and capsorubin in the storage roots of WT and transgenic plants, respectively. **m** Total carotenoid content of WT and transgenic plants. Error bars indicate SD (*n* = 3). ^*^ and ^**^ indicate a significant difference from that of WT at *P* < 0.05 and *P* < 0.01, respectively, by Student’s *t*-test.

To further investigate whether *IbNAC29* regulates carotenoids in sweet potato, we generated *IbNAC29*-overexpression (*IbNAC29*-OE) plants by *Agrobacterium*-mediated transformation of sweet potato variety Lizixiang (Figs S1 and S2, see online supplementary material). After examining the *IbNAC29* mRNA levels in these plants using qRT-PCR, we selected three lines (OE-2, OE-7, and OE-23) with the up-regulated *IbNAC29* mRNA levels for further study (Fig. S1, see online supplementary material). Cross-sectional flesh samples of the transgenic lines were slightly yellower and had orange spots relative to the wild type (WT) (Fig. 3a).

Because carotenoids are stored in plastids [30, 31], we next analysed the plastids in the storage roots of transgenic *IbNAC29*-OE using transmission electron microscopy (TEM). The number of carotenoid globules in the *IbNAC29*-OE plants was significantly increased compared with the WT (Fig. 3a), suggesting the high levels of carotenoids accumulation in the storage roots of *IbNAC29*-OE plants.

Sweet potato contains various carotenoids, including α-carotene, lutein, β-carotene, zeaxanthin, capsanthin, violaxanthin, β-cryptoxanthin, echinenone, neoxanthin, antheraxanthin, and capsorubin. Next, we determined the concentration of different carotenoids in the storage roots of *IbNAC29*-OE and WT plants. We found that the levels of α-carotene (0.0328–0.0403 μg/g DW), lutein (0.1816–0.2212 μg/g DW), β-carotene (0.1512–0.2888 μg/g DW), zeaxanthin (0.1081–0.1558 μg/g DW), capsanthin (0.0082–0.0094 μg/g DW), and β-cryptoxanthin (0.8637–1.001 μg/g DW) are significantly increased, respectively, while the level of violaxanthin (0.0023–0.0040 μg/g DW) is decreased in the *IbNAC29*-OE plants (Fig. 3b–l). There is no significant difference in the level of capsorubin between IbNAC29-OE and WT plants. Eventually, total carotenoid content is significantly increased in the storage roots of transgenic plants compared with WT (Fig. 3m).

### Carotenoid biosynthesis-related genes are upregulated in *IbNAC29*-OE plants

**Figure 4:**
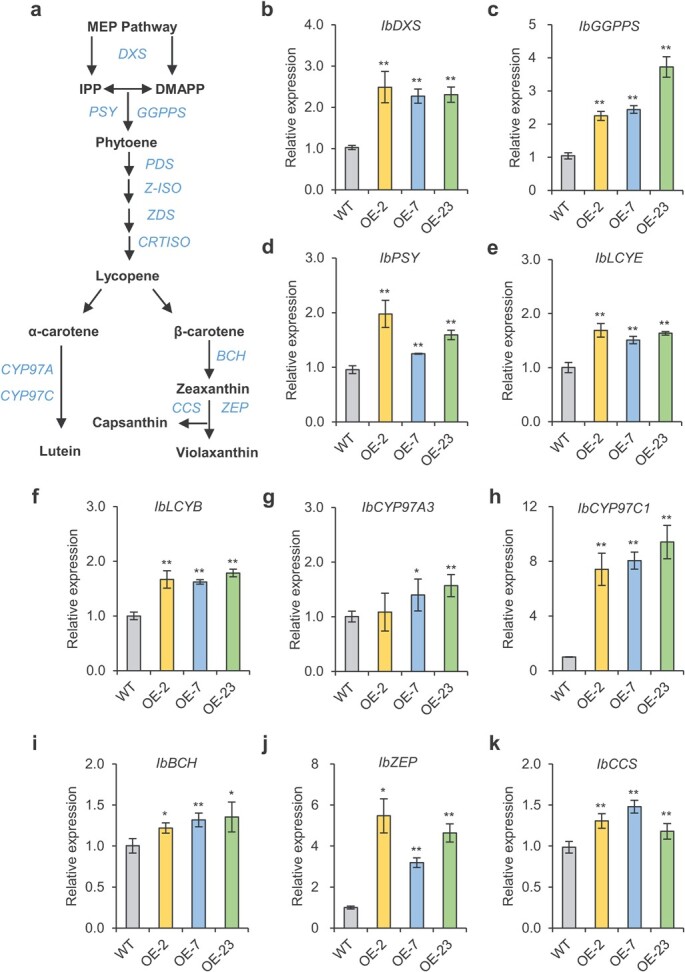
Carotenoid biosynthetic pathway and expression levels of carotenoid biosynthetic-related genes in the storage roots of *IbNAC29*-OE plants. **a** General carotenoid biosynthetic pathway in plants. **b** MEP pathway gene, *IbDXS,* for carotenoid precursor supply. **c**–**f** Carotene biosynthetic genes, including *IbGGPPS*, *IbPSY*, *IbLCYE,* and *IbLCYB*. **g**–**k** Xanthophyll biosynthetic genes, including *IbCYP97A3*, *IbCYP97C1*, *IbBCH*, *IbZEP,* and *IbCCS*. *IbActin* was used as the internal control. The transcript level in WT was set as control. Error bars indicate SD (*n* = 3). ^*^ and ^**^ indicate a significant difference from that of WT at *P* < 0.05 and *P* < 0.01, respectively, by Student’s *t*-test.

Next, we used qRT-PCR assays to determine the expression of carotenoid biosynthesis-related genes in *IbNAC29*-OE plants and WT at storage root expansion stage. The carotenoid biosynthesis pathway is shown in Fig. 4a. In this study, we observed elevated mRNA levels of *IbDXS*, one MEP pathway gene (Fig. 4b) and four carotene biosynthesis genes (*IbGGPPS*, *IbPSY*, *IbLCYE*, and *IbLCYB*) (Fig. 4c–f).

Previous research has shown that CYP97A (cytochrome P450 monooxygenase) works synergistically with CYP97C to hydroxylate α-carotene into lutein [32–34]. Both *IbCYP97A3* and *IbCYP97C1* are elevated in IbNAC29-OE*.* Therefore, the upregulated *IbCYP97A3* and *IbCYP97C1* might lead to the lutein accumulation in *IbNAC29*-OE plants (Fig. 3c and 4g–h).

Interestingly, we found increased zeaxanthin and capsanthin levels, but a decreased violaxanthin level. The expression of *IbBCH*, *IbZEP*, and *IbCCS* was also activated in *IbNAC29*-OE. We thus proposed that the decreased violaxanthin level may be because of its conversion to capsanthin under the high *IbCCS* expression (Fig. 3e–g and 4i–k). Therefore, our results suggested that the upregulation of carotenoid biosynthesis genes causes the carotenoid accumulation in the storage roots of transgenic *IbNAC29*-OE sweet potato.

### IbNAC29 could not bind to the promoters of carotenoid biosynthesis-related genes

When *IbNAC29* was overexpressed in the sweet potato, the genes for carotenoid biosynthesis were significantly elevated in *IbNAC29*-OE. Next, we performed the yeast one-hybrid (Y1H) experiment to investigate the potential relationship of IbNAC29 and the promoters of the above genes. The promoter fragments of *IbGGPPS*, *IbPSY*, *IbLCYE*, and *IbLCYB* were independently amplified by PCR using genomic DNA as the template and cloned into the pLacZi2μ vector. The yeast activation domain (AD) was fused with the coding sequence of *IbNAC29* to form the effector 42 AD-IbNAC29 construct. Both the reporter constructs and the effector 42 AD-IbNAC29 were cotransformed into yeast; 42 AD alone as a negative control. As shown in Fig. S4 (see online supplementary material), IbNAC29 protein did not bind to these promoters. These results remind us that IbNAC29 may indirectly influence carotenoid biosynthesis via other factors.

### IbNAC29 forms a regulatory module with IbMYB1R1 and IbAITR5

**Figure 5:**
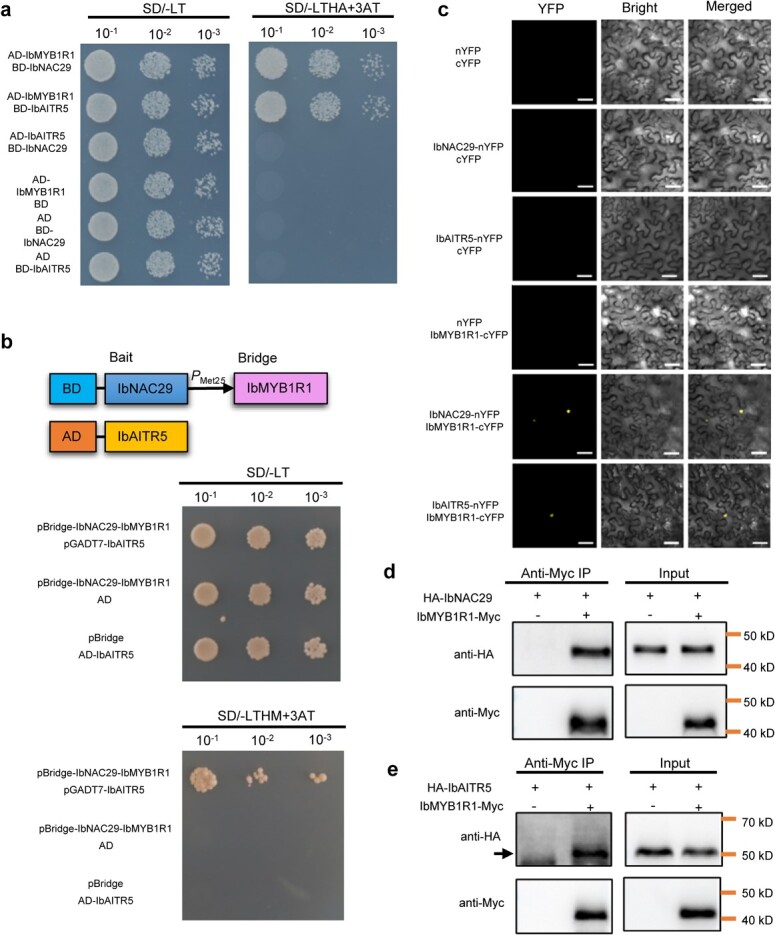
Interactions between IbNAC29, IbMYB1R1, and IbAITR5. **a** Interactions among IbNAC29, IbMYB1R1, and IbAITR5 by Y2H assays. **b** Y3H assays detected the interactions between IbNAC29, IbMYB1R1, and IbAITR5. **c** Confirmation of the interaction between IbNAC29 and IbMYB1R1, IbMYB1R1 and IbAITR5 by BiFC, as indicated by the yellow fluorescent signal. Bar = 50 μm. **d** and **e** Co-IP assays showing that IbMYB1R1 interacts with IbNAC29 (**d**) and IbAITR5 (**e**) *in vivo*. Total proteins from *Nicotiana benthamiana* leaf cells expressing IbMYB1R1-Myc, HA-IbNC29, and HA-IbAITR5 were extracted and incubated with anti-Myc magnetic beads. Total extracts before (input) and after IP were detected with anti-HA and anti-Myc antibodies.

To investigate the possible interacting partners of IbNAC29 involved in carotenoid biosynthesis, we screened the sweet potato yeast two-hybrid (Y2H) library. Among these potential interacting proteins, we identified an R1-type MYB1 protein IbMYB1R1. Previous studies have shown that R2R3-type MYB, along with other factors, form a regulatory complex which affects anthocyanin biosynthesis [21, 35, 36]. Through yeast two-hybrid library screening, we isolated a IbMYB1R1-interacting protein IbAITR5. IbAITR5 belongs to a novel family of transcription factors, working as a member of ABA-induced transcription repressors (AITRs). The Y2H assays revealed that although IbNAC29 and IbAITR5 individually interacted with IbMYB1R1, there was no interaction between IbNAC29 and IbAITR5 (Fig. 5a). Using the yeast three-hybrid (Y3H) assays, we also observed that IbNAC29, IbMYB1R1, and IbAITR5 apparently formed a regulatory module (Fig. 5b). These interactions among IbNAC29, IbMYB1R1, and IbAITR5 were verified in the leaf epidermal cells of *Nicotiana benthamiana* using bimolecular fluorescence complementation (BiFC) assays. We observed a sharp yellow fluorescence in the nucleus when IbNAC29-nYFP or IbAITR5-nYFP was co-expressed with IbMYB1R1-cYFP, while negative controls showed no YFP fluorescence signal (Fig. 5c). Furthermore, we found that the IbMYB1R1 and IbAITR5 proteins were localized in the nuclei of the protoplasts (Fig. S5, see online supplementary material), which was consistent with the location of IbNAC29, thereby suggesting that IbNAC29, IbMYB1R1, and IbMYB1R1 may form a regulatory module and function in the nucleus.

Next, we used co-immunoprecipitation (co-IP) assays to investigate the IbNAC29-IbMYB1R1 and IbMYB1R1-IbAITR5 interactions *in vivo*. We isolated the total proteins co-expressed by IbMYB1R1-Myc with HA-IbNAC29 or HA-IbAITR5 in the leaf epidermal cells of *Nicotiana benthamiana*, and incubated them with anti-c-Myc agarose beads. We detected HA-IbNAC29 and HA-IbAITR5 in the immunoprecipitated proteins, but not in the negative control (Fig. 5d–e). These experiments further indicated that IbMYB1R1 physically interacts with IbNAC29 and IbAITR5 in planta*,* confirming the previous results.

Taken together, these results confirmed that IbNAC29 could interact with IbMYB1R1, which forms an intermediate bridge with IbAITR5 to potentially form the IbNAC29-IbMYB1R1-IbAITR5 regulatory module.

### IbAITR5 directly binds to the *IbSGR1* promoter and represses its transcript activity

We first examined the relative mRNA level of the *SGR1*-homologous gene *IbSGR1* in *IbNAC29*-OE plants using qRT-PCR. qRT-PCR analysis revealed that relative *IbSGR1* mRNA level was strongly reduced in the *IbNAC29*-OE plants (Fig. S6a, see online supplementary material), suggesting that *IbNAC29* may negatively regulate *IbSGR1*.

**Figure 6:**
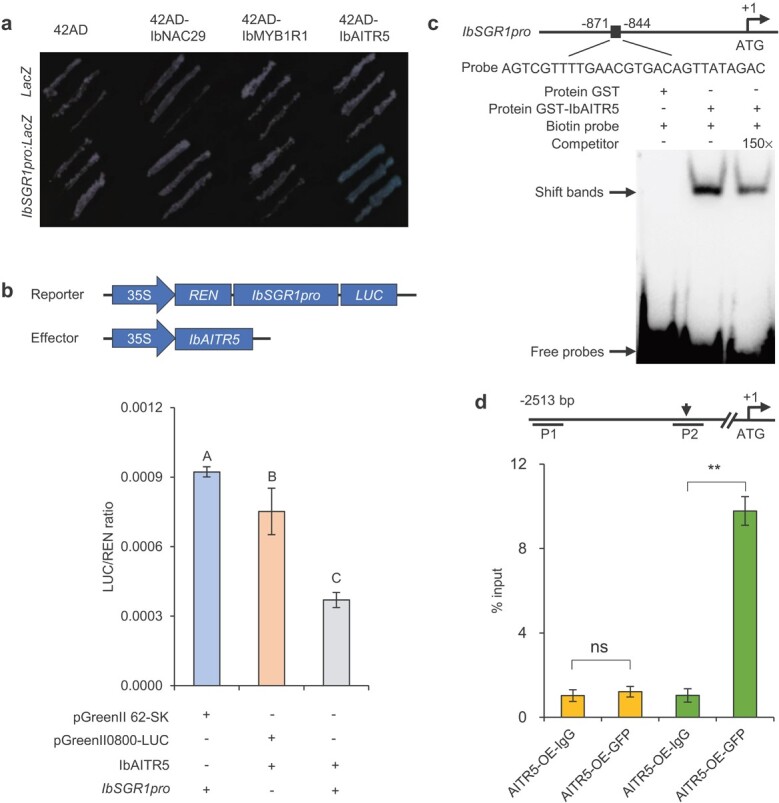
Interactions of IbAITR5 with the *IbSGR1* promoter. **a** Y1H assay showing that IbAITR5 binds to the promoter of *IbSGR1*. Yeast cells containing *IbSGR1pro:LacZ* were transformed with IbNAC29, IbMYB1R1, and IbAITR5 fused with the 42 AD and grown on medium containing X-Gal. Coexpression of 42 AD/LacZ, 42 AD- IbNAC29/LacZ, 42 AD- IbMYB1R1/LacZ, 42 AD-IbAITR5/LacZ, and 42 AD/*IbSGR1pro:LacZ* was used as the negative controls. **b** IbAITR5 inhibited the promoter activity of *IbSGR1* determined by the dual-luciferase assays in protoplasts. Relative activity of the *IbSGR1* promoter was represented by the LUC/REN ratio. + and − indicated presence and absence, respectively. Error bars indicate SD (*n* = 4). Ordinary one-way ANOVA multiple comparison, with different letters indicating the statistically significant differences at *P* < 0.01. **c** EMSA showing that IbAITR5 binds to an NACRS element of the *IbSGR1* promoter. The recombinant IbAITR5-GST protein retarded the shift of the labelled probes; 150× indicated adding excess non-labelled probes as competitors. + and − indicated presence and absence, respectively. **d** ChIP-qPCR analysis showed IbAITR5 could bind to the *IbSGR1* promoter in the chromatin immunoprecipitated with an anti-GFP antibody from the 35S:IbAITR5-GFP plants. AITR5-OE-IgG, no antibody control samples. The NACRS element in segment P2 was represented by an arrow. Segment P1 was used as the negative control. Error bars indicate SD (*n* = 4). ns, no significance. ^**^P < 0.01, as determined by Student’s *t*-test analysis.

To test the hypothesis, we conducted Y1H assays to explore the relationship between the IbNAC29-IbMYB1R1-IbAITR5 regulatory module and the *IbSGR1* promoter. Interestingly, we found that IbAITR5, rather than IbNAC29 and IbMYB1R1, directly binds to the *IbSGR1* promoter (Fig. 6a). Then, we used the dual-luciferase reporter assays to assess the luciferase activity of *IbSGR1* driven by the IbAITR5. These results revealed that when *IbSGR1pro:LUC* was co-transformed with IbAITR5, IbAITR5 inhibited the *IbSGR1* promoter activity. Therefore, our data demonstrated that IbAITR5 represses the *IbSGR1* promoter activity by binding to its promoter (Fig. 6b).

Next, we used the electrophoretic mobility shift assay (EMSA) and chromatin immunoprecipitation-quantitative polymerase chain reaction (ChIP-qPCR) assays to validate whether IbAITR5 could bind to the *IbSGR1* promoter. In the EMSA assay, IbAITR5-GST bound to a 28 bp fragment of *IbSGR1in vitro* (Fig. 6c). Additionally, the ChIP-qPCR assays confirmed that IbAITR5 also binds *in vivo* to the *IbSGR1* promoter (Fig. 6d). Thus, our results collectively suggested that IbAITR5 represses *IbSGR1* transcription by directly binding to its promoter.

### IbNAC29-IbMYB1R1-IbAITR5 regulatory module regulates carotenoid biosynthesis

**Figure 7:**
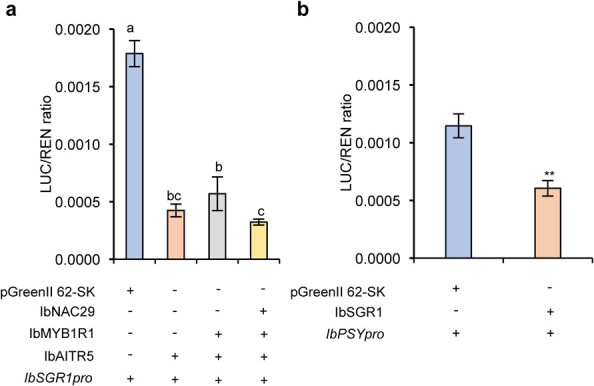
Effects of IbNAC29, IbMYB1R1, and IbAITR5 and their complexes on downstream genes. **a** IbNAC29 enhanced the inhibitory activity of IbAITR5 on the downstream *IbSGR1pro* via IbMYB1R1 in the protoplasts. + and − indicated presence and absence, respectively. Error bars indicate SD (*n* = 4). Ordinary one-way ANOVA multiple comparison, with different letters indicating the statistically significant differences at *P* < 0.05. **b** IbSGR1 inhibited the *IbPSY* promoter activity in protoplasts. + and − indicated presence and absence, respectively. Error bars indicate SD (*n* = 4). Ordinary one-way ANOVA multiple comparison, with different letters indicating statistically significant differences at *P* < 0.01.

To further investigate how IbNAC29, IbMYB1R1, and IbAITR5 affected the transcriptional activity of *IbSGR1*, we conducted the dual-luciferase reporter assays. As shown in Fig. 7a, the luciferase activity remained unchanged when IbMYB1R1 vector co-transient with IbAITR5 and *IbSGR1pro* vectors compared with IbAITR5 and *IbSGR1pro* vector co-transient in protoplasts. However, in the presence of IbMYB1R1, IbNAC29 enhanced the inhibitory activity of IbAITR5 on the *IbSGR1* promoter (Fig. 7a).

It has been reported that SlSGR1 influences the *SlPSY1* expression pattern in tomato [24]. Furthermore, the dual-luciferase assays revealed that the IbSGR1 also influences the expression of *IbPSY* (Fig. 7b). The repression of the *IbPSY1* gene in the presence of *IbSGR1* expression is in accordance with previous studies [24]. Therefore, our results suggest that the IbNAC29-IbMYB1R1-IbAITR5 regulatory module potentially regulates carotenoid biosynthesis via the regulation of *IbPSY1*.

## Discussion

Carotenoids are tetraterpenoids molecules that play pivotal roles in photosynthesis, pigmentation, and development. Despite an in-depth mechanistic basis for understanding the carotenoid biosynthesis, relatively little is known about how this pathway is transcriptionally regulated. Previously, we conducted the transcriptome database of differentially expressed genes between the Weiduoli and its high-carotenoid mutant HVB-3 [27]. Among these genes, NAC transcription factors *IbNAC29*, *IbNAC74*, and *IbNAC87* were upregulated in HVB-3. In this study, we selected and characterized *IbNAC29* gene. Transgenic experiments demonstrated overexpression of *IbNAC29* increased the levels of various carotenoids in the storage roots, including α-carotene, lutein, β-carotene, zeaxanthin, and capsanthin (Fig. 3).

Indeed, the carotenoid biosynthetic gene expression (*IbDXS*, *IbGGPS*, *IbPSY,* etc) was also up-regulated in *IbNAC29* transgenic plants. This could potentially explain why carotenoid accumulation is elevated. Previous reports have suggested that overexpression of *PmDXS* and *IbGGPS* increased the carotenoid content in *Arabidopsis* [37, 38]. Furthermore, overexpressing *LCYE* elevates the carotenoid lutein level in *Arabidopsis* leaves [39]. Also, overexpression of *IbLCYB2* increases the carotenoid content in the sweet potato’s storage roots [40]. In plants, the *SGR* gene encodes the key enzyme for chlorophyll degradation [23]. In tomato, *SlSGR1* reportedly regulates chlorophyll degradation [22, 24]. Silencing *SlSGR1* inhibits chlorophyll degradation, resulting in the retention of a green phenotype. As a matter of fact, *SlSGR1* regulates the lycopene accumulation in tomato by directly inhibiting the activity of a key carotenoid biosynthesis enzyme, SlPSY1 [24]. Overexpression of *CsPSY* enhances carotenoid accumulation in Hongkong kumquat [41]. Both CsSGRa and CsSGRb interact with CsPSY1 to inhibit the citrus carotenoid biosynthesis, chlorophyll degradation and carotenoid biosynthesis, which are highly conserved processes in plants [42]. Similarly, the overexpression of *CsPSY* enhances carotenoid accumulation in Hongkong kumquat [41]. Therefore, our result suggested that the upregulation of carotenoid biosynthesis genes might cause the accumulation in the carotenoids.

Previous studies have reported that the tomato NAC transcription factor SlNOR-like1 directly binds to the *SGR1* promoter, thus regulating fruit ripening and carotenoid accumulation [16]. However, Y1H assay indicated IbNAC29 could not directly bind to the promoters of carotenoid biosynthesis-related enzymes. To explore the possible mechanism of IbNAC29 involved in carotenoid biosynthesis, we screened the sweet potato yeast two-hybrid (Y2H) library. Among these potential interacting proteins, we identified an R1-type MYB1 protein IbMYB1R1. Previous studies have shown that R2R3-type MYB, along with other factors, form a regulatory complex which affects anthocyanin biosynthesis [21, 35, 36]. Through yeast two-hybrid library screening, we isolated a IbMYB1R1-interacting protein IbAITR5. In our study, the results showed that IbAITR5 could directly bind to the *IbSGR1* promoter, inhibiting the expression of the *IbSGR1* (Fig. 6). The mRNA level of *IbSGR1* is down-regulated in IbNAC29-OE, which is consistent with its negative role in carotenoid accumulation. Although we detected enhanced carotenoids accumulation in the *IbNAC29*-OE storage roots (Fig. 3), we did not find any direct interaction between *IbNAC29* and the *IbSGR1* promoter (Fig. 6a). Therefore, our results suggested that IbNAC29 might have a different regulatory mechanism with SlNOR-like1, possibly because they belong to different clades in the evolutionary tree.

**Figure 8:**
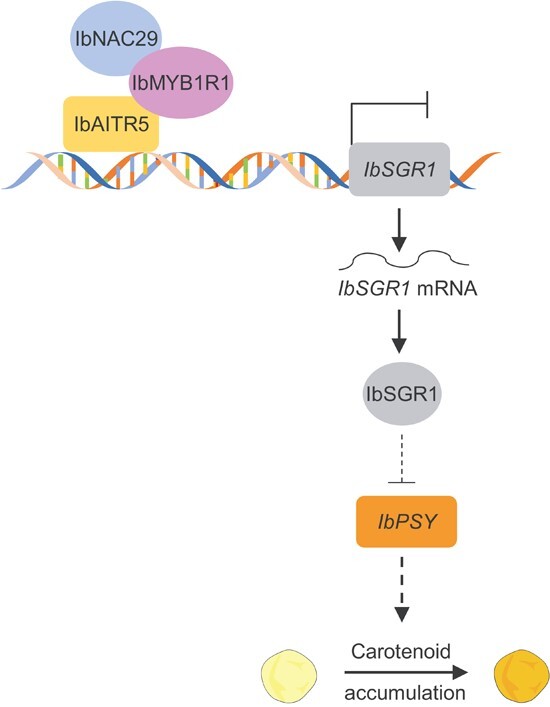
Proposed model of how *IbNAC29* regulates carotenoid biosynthesis. IbNAC29, IbMYB1R1, and IbAITR5 form a regulatory module. IbAITR5 binds to and represses the promoter activity of *IbSGR1*. Elevated levels of *IbNAC29* enhance the IbAITR5-mediated inhibition of IbSGR1 activity, reducing the inhibition of *IbPSY* gene expression and increasing the accumulation of carotenoids.

Through Y3H, EMSA, ChIP-qPCR, and dual-luciferase assay analyses, our study demonstrated that the IbNAC29-IbMYB1R1-IbAITR5 regulatory module mediates the carotenoids biosynthesis via protein–protein interactions to regulate the downstream target gene expression in sweet potato. It has been reported that AITRs are transcription repressors in plants [43], and we found that the *IbAITR5* mRNA level in the *IbNAC29*-OE plants was upregulated (Fig. S6b, see online supplementary material). Thus, we proposed that *IbNAC29* enhances the inhibitory activity of IbAITR5 by affecting its transcriptional activity. This leads to reduce the expression of the *IbSGR1* (Figs 2a and 7a), resulting in further alleviation of the inhibition of IbSGR1 on mRNA level of the key carotene biosynthesis gene *IbPSY*. Up-regulated expression of *IbPSY* might lead to enhanced carotenoids accumulation in the storage roots (Fig. 8).

Altogether, our findings unveil the mechanism underlying the regulation of the carotenoids accumulation and provide new insights for genetic improvement in the sweet potato. To further understand the mechanisms that regulate carotenoid biosynthesis in staple crops, we will further identify the direct targets of *IbNAC29* by combining transcriptome analysis with chromatin immunoprecipitation analysis in the future. Moreover, we will attempt to use the CRISPR/Cas9-based gene editing approach to further understand its role in the development of sweet potato.

## Materials and methods

### Plant materials and growth conditions

Sweet potato cultivar Weiduoli with orange-flesh and its high carotenoid mutant HVB-3 were used for RNA sequencing analyses. Sweet potato cultivar Lizixiang was used as the recipient for *Agrobacterium*-mediated transformation, which is a pale-yellow flesh with low carotenoid content. Transgenic test-tube seedlings were grown on Murashige and Skoog medium at 28°C with a 13-h-light/11-h-dark cycle. The transgenic plants were cultivated in the field of the experimental stations of China Agricultural University and adhered to normal agricultural practice.

### Gene identification and sequence analysis

Total RNA was extracted using TRIzol reagent (Invitrogen, USA). Complementary DNAs (cDNA) were obtained using HiFiScript gDNA Removal cDNA Synthesis Kit (CwBio, Beijing, China) according to the manufacturer’s protocol. The RACE (rapid amplification of cDNA ends) experiment was used to obtain the full-length cDNA sequence of *IbNAC29*. According to the EST sequence obtained from previous studies [27], the coding sequences of *IbMYB1R1, IbAITR5,* and *IbSGR1* were obtained from Lizixiang using the homologous cloning method. DNAMAN software, MEGA 7.0 software, and the Splign tool were used to analyse amino acid sequence alignments, exon-intron, and phylogenetic relationships, respectively.

### Subcellular localization analysis

The open reading frames of *IbNAC29*, *IbMYB1R1*, and *IbAITR5* without the stop codon were inserted into the pCAMBIA1300-35S-GFP vector. The recombinant vector pBI121-ARF-mCherry containing a nuclear marker ARF1 was co-transformed with pCAMBIA1300-35S-IbNAC29-GFP, pCAMBIA1300-35S-IbMYB1R1-GFP, and pCAMBIA1300-35S-IbAITR5-GFP, respectively. Meanwhile, pCAMBIA1300-35S-GFP and pBI121-35S-ARF1-mCherry were co-transformed into protoplasts as a control. After growing for 16 h, the fluorescence signals of GFP and mCherry were visualized by a confocal fluorescence microscopy (Olympus, Tokyo, Japan) under excitation wavelengths of 488 nm and 546 nm, respectively.

### Sweet potato transformation and qRT-PCR analysis

The embryogenic suspension cultures of Lizixiang were transformed with the pCAMBIA1300-35S-IbNAC29-GFP vector via *Agrobacterium-*mediated transformation [44]. The transgenic sweet potato plants were selected using hygromycin as a selection marker. The plants were transferred to a greenhouse, planted in the nutrient vegetative soil, and then transplanted to the field for phenotype observation. The *IbActin* gene of sweet potato (*AY905538*) was used as the internal control for expression analysis by qRT-PCR assays [45, 46]. The mRNA levels of genes were calculated by comparative CT method [47]. The experiment was conducted using three biological replicates consisting of pools of three plants. Values are means ± SD of three biological repeats.

### Measurement of carotenoid contents

Carotenoids were extracted as described previously [37]. Three independent storage roots from each freshly harvested WT and *IbNAC29*-OE transgenic plant were mixed, respectively. Carotenoids and the relative contents were measured as previously described [48].

### Transmission electron microscope

The storage roots of *IbNAC29*-OE and WT were fixed as previously described [40]. The number of carotenoid globules was observed using TEM (JEM-1230, Tokyo, Japan).

### Yeast assays

In the Y1H assay, the open reading frames of *IbNAC29*, *IbMYB1R1*, and *IbAITR5* sequences were separately cloned into the pB42AD vector. The promoter sequences of *IbGGPPS*, *IbPSY*, *IbLCYB*, *IbLCYE*, and *IbSGR1* genes from Lizixiang were cloned separately into the pLacZi2μ vector. In short, various LacZ reporter plasmids were cotransformed with the pB42AD fusion constructs into EGY48 yeast strain. The pLacZi2μ reporter and pB42AD were co-transformed as negative controls. Transformants were grown on SD/−Trp-Ura dropout plates containing 5-bromo-4-chloro-3-indolyl-β-D-galactopyranoside (X-Gal) for blue color development.

Y2H assay was done according to the Matchmaker™ Gold Yeast Two-Hybrid System User Manual (Clontech). The coding sequences of *IbNAC29*, *IbMYB1R1*, and *IbAITR5* were cloned into either the bait vector pGBKT7 or the prey vector pGADT7. Transformed Y2H-Gold yeast cells were patched onto the SD/−Leu/−Trp (SD/−LT) and SD/−Leu/−Trp/-His/−Ade (SD/−LTHA) +6 mM 3AT plates and grown at 30°C.

Y3H assay was conducted as previously described [49]. The open reading frames of *IbNAC29* and *IbMYB1R1* were cloned into the pBridge vector, while the coding sequence of *IbAITR5* was cloned into the pGADT7 vector. The combinations of pBridge-IbNAC29-IbMYB1R1 with pGADT7-IbAITR5, pBridge-IbNAC29-IbMYB1R1 with pGADT7, and pBridge with pGADT7-IbAITR5 were co-transformed into yeast. The combinations containing the empty pBridge or pGADT7 vectors were used as negative controls. Transformed Y2H-Gold yeast cells were patched on the SD/−Leu/−Trp (SD/−LT) and SD/−Leu/−Trp/-His/−Met (SD/−LTHM) +6 mM 3AT plates and grown at 30°C.

### BiFC assay

Empty pSPYNE-35S or the pSPYCE-35S vector cloned with the *IbNAC29*, *IbMYB1R1*, and *IbAITR5* coding sequences were transformed into the *Agrobacterium tumefaciens* strain EHA105. Combinations of pSPYNE and pSPYCE vectors, together with P19, were infiltrated into the *Nicotiana benthamiana* leaf epidermal cells. The YFP signal was observed by using a laser confocal scanning microscope at an excitation wavelength of 488 nm after 48 h growth (Olympus, Tokyo, Japan).

### Co-IP assay

Co-IP assay was performed as mentioned previously [46]. The anti-HA primary antibody (MilliporeSigma), anti-Myc primary antibody (MilliporeSigma), Goat anti-mouse IgG secondary antibody (Light chain specific, Easybio), and Anti-c-Myc agarose beads (MilliporeSigma) were used to detect samples.

### Dual-luciferase assay

Rice shoot protoplasts were isolated and used for the dual-luciferase assays, as described previously [50]. For the transcriptional activity assay, the empty pBD vector was used as the negative control to measure the transcriptional activity of *IbNAC29*.

For the DNA-promoter interaction assay, the *IbNAC29*, *IbMYB1R1*, *IbAITR5,* and *IbSGR1* coding sequences were cloned separately into the pGreenII 62-SK vector. The *IbSGR1* and *IbPSY* promoters were cloned separately into the pGreenII0800-LUC vector. Firefly luciferase (LUC) and Renilla luciferase (REN) activity levels were measured using a dual-luciferase reporter assay system (Promega, USA). Four technical replicates were conducted in the experiments.

### EMSA

EMSA was performed according to the manufacturer’s instructions (Thermo Fisher Scientific, USA). Glutathione beads purified recombinant GST-labeled IbAITR5 protein expressed in *Escherichia coli* Transetta (DE3). The NACRS element containing biotin-labeled probes synthesized by Tsingke (Beijing) were used as binding probes, while unlabeled probes were used as competing probes.

### ChIP-qPCR analysis

The ChIP assay was carried out as described previously [46]. The plants of pSuper1300-IbAITR5-GFP were cut into pieces and immediately fixed with 1% (v/v) formaldehyde solution. Next, the samples were ground into fine powders under liquid nitrogen. StepOnePlus™ was used to analyse the enrichment of immunoprecipitated DNA. *IbSGR1* promoter P2 fragment contained a NACRS element (sequence is ACGTGA), while P1 having no NACRS element served as the negative control. Four technical replicates were conducted in the experiments using. All the above primer sequences are shown in Table S1 (see online supplementary material).

#### Accession numbers

Sequence data from this article can be found in the Sweet Potato Genomics Resource database (http://sweetpotato.uga.edu) under accession numbers *IbNAC29* (itf01g25900.t1), *IbSGR1* (itf08g00520.t1), *IbMYB1R1* (itf03g18010.t1), *IbAITR5* (itf11g06190.t1), *IbGGPPS* (itf08g03960.t1), *IbPSY* (itf03g05110.t1), *IbLCYE* (itf12g20540.t1), and *IbLCYB* (itf01g24560.t1).

## Acknowledgements

This study was supported by the National Natural Science Foundation of China (31872878) and the earmarked fund for CARS-10-Sweetpotato.

## Author contributions

S.X. and Q.L. designed the experiments. S.X., R.L., H.Zhao, H.Zhai, H. Zhang, S.H., Y.Z., and N.Z. performed the experiments. S.X., R.L., Y.Z., H. Zhao, and S.G. analysed the data. S.X. drafted the manuscript. S.G. and Q.L. revised and finalized the manuscript. All authors discussed the results and approved the final article.

## Data availability

The data supporting the findings of this work are available within the paper and its online supplementary material.

## Conflict of interest

None declared.

## Supplementary data

Supplementary data is available at *Horticulture Research* online.

## Supplementary Material

Web_Material_uhad010

## References

[1] McQuinn RP , Gapper NE, Gray AG et al. Manipulation of ZDS in tomato exposes carotenoid-and ABA-specific effects on fruit development and ripening. Plant Biotechnol J. 2020;18:2210–24. 32171044 10.1111/pbi.13377 PMC7589306

[2] Johnson JD . Do carotenoids serve as transmembrane radical channels? Free Radical Bio Med. 2009;47:321–3. 19446633 10.1016/j.freeradbiomed.2009.05.008

[3] Nisar N , Li L, Lu S et al. Carotenoid metabolism in plants. Mol Plant. 2015;8:68–82. 25578273 10.1016/j.molp.2014.12.007

[4] Krinsky NI , Johnson EJ. Carotenoid actions and their relation to health and disease. Mol Asp Med. 2005;26:459–516. 10.1016/j.mam.2005.10.001 16309738

[5] Fraser PD , Bramley PM. The biosynthesis and nutritional uses of carotenoids. Prog Lipid Res. 2004;43:228–65. 15003396 10.1016/j.plipres.2003.10.002

[6] Pulido P , Toledo-Ortiz G, Phillips MA et al. Arabidopsis J-protein J20 delivers the first enzyme of the plastidial isoprenoid pathway to protein quality control. Plant Cell. 2013;25:4183–94. 24104567 10.1105/tpc.113.113001 PMC3877790

[7] Cazzonelli CI , Pogson BJ. Source to sink: regulation of carotenoid biosynthesis in plants. Trends Plant Sci. 2010;15:266–74. 20303820 10.1016/j.tplants.2010.02.003

[8] Farré G , Sanahuja G, Naqvi S et al. Travel advice on the road to carotenoids in plants. Plant Sci. 2010;179:28–48.

[9] Kang L , Park SC, Ji CY et al. Metabolic engineering of carotenoids in transgenic sweetpotato. Breed Sci. 2017;67:27–34. 28465665 10.1270/jsbbs.16118 PMC5407916

[10] Ku H-K , Jeong YS, You MK et al. Alteration of carotenoid metabolic machinery by β-carotene biofortification in rice grains. J Plant Biol. 2019;62:451–62.

[11] Jeknić Z , Morré JT, Jeknić S et al. Cloning and functional characterization of a gene for capsanthin-capsorubin synthase from tiger lily (*Lilium lancifolium* Thunb. ‘Splendens’). Plant Cell Physiol. 2012;53:1899–912. 23008421 10.1093/pcp/pcs128 PMC3494009

[12] Guzman I , Hamby S, Romero J et al. Variability of carotenoid biosynthesis in orange colored *capsicum spp*. Plant Sci. 2010;179:49–59. 20582146 10.1016/j.plantsci.2010.04.014 PMC2889374

[13] Kou X , Zhao Y, Wu C et al. SNAC4 and SNAC9 transcription factors show contrasting effects on tomato carotenoids biosynthesis and softening. Postharvest Biol Tec. 2018;144:9–19.

[14] Ma N , Feng H, Meng X et al. Overexpression of tomato *SlNAC1* transcription factor alters fruit pigmentation and softening. BMC Plant Biol. 2014;14:351. 25491370 10.1186/s12870-014-0351-y PMC4272553

[15] Zhu M , Chen G, Zhou S et al. A new tomato NAC (NAM/ATAF1/2/CUC2) transcription factor, SlNAC4, functions as a positive regulator of fruit ripening and carotenoid accumulation. Plant Cell Physiol. 2014;55:119–35. 24265273 10.1093/pcp/pct162

[16] Gao Y , Wei W, Zhao X et al. A NAC transcription factor, NOR-like1, is a new positive regulator of tomato fruit ripening. Hortic Res. 2018;5:75. 30588320 10.1038/s41438-018-0111-5 PMC6303401

[17] Gao Y , Wei W, Fan Z et al. Re-evaluation of the nor mutation and the role of the NAC-NOR transcription factor in tomato fruit ripening. J Exp Bot. 2020;71:3560–74. 32338291 10.1093/jxb/eraa131 PMC7307841

[18] Aharoni A , de Vos CHR, Wein M et al. The strawberry FaMYB1 transcription factor suppresses anthocyanin and flavonol accumulation in transgenic tobacco. Plant J. 2001;28:319–32. 11722774 10.1046/j.1365-313x.2001.01154.x

[19] Sagawa JM , Stanley LE, LaFountain AM et al. An R2R3-MYB transcription factor regulates carotenoid pigmentation in *Mimulus lewisii* flowers. New Phytol. 2016;209:1049–57. 26377817 10.1111/nph.13647

[20] Zhu F , Luo T, Liu C et al. An R2R3-MYB transcription factor represses the transformation of α- and β-branch carotenoids by negatively regulating expression of *CrBCH2* and *CrNCED5* in flavedo of *citrus reticulate*. New Phytol. 2017;216:178–92. 28681945 10.1111/nph.14684

[21] Meng Y , Wang Z, Wang Y et al. The MYB activator WHITE PETAL1 associates with MtTT8 and MtWD40-1 to regulate carotenoid-derived flower pigmentation in *Medicago truncatula*. Plant Cell. 2019;31:2751–67. 31530734 10.1105/tpc.19.00480 PMC6881138

[22] Hörtensteiner S . Stay-green regulates chlorophyll and chlorophyll-binding protein degradation during senescence. Trends Plant Sci. 2009;14:155–62. 19237309 10.1016/j.tplants.2009.01.002

[23] Thomas H , Howarth CJ. Five ways to stay green. J Exp Bot. 2000;51:329–37. 10938840 10.1093/jexbot/51.suppl_1.329

[24] Luo Z , Zhang J, Li J et al. A STAY-GREEN protein SlSGR1 regulates lycopene and β-carotene accumulation by interacting directly with SlPSY1 during ripening processes in tomato. New Phytol. 2013;198:442–52. 23406468 10.1111/nph.12175

[25] Xiao Y , Zhu M, Gao S. Genetic and genomic research on sweet potato for sustainable food and nutritional security. Genes. 2022;13:1833. 36292718 10.3390/genes13101833 PMC9602178

[26] Teow CC , Truong VD, McFeeters RF et al. Antioxidant activities, phenolic and β-carotene contents of sweet potato genotypes with varying flesh colours. Food Chem. 2007;103:829–38.

[27] Li R , Zhai H, Kang C et al. De novo transcriptome sequencing of the orange-fleshed sweet potato and analysis of differentially expressed genes related to carotenoid biosynthesis. Int J Genomics. 2015;2015:843802. 26649293 10.1155/2015/843802 PMC4663004

[28] Marchler-Bauer A , Derbyshire MK, Gonzales NR et al. CDD: NCBI's conserved domain database. Nucleic Acids Res. 2015;43:D222–6. 25414356 10.1093/nar/gku1221 PMC4383992

[29] Waller F , Furuya M, Nick P. OsARF1, an auxin response factor from rice, is auxin-regulated and classifies as a primary auxin responsive gene. Plant Mol Biol. 2002;50:415–25. 12369618 10.1023/a:1019818110761

[30] Li L , Yuan H. Chromoplast biogenesis and carotenoid accumulation. Arch Biochem Biophys. 2013;539:102–9. 23851381 10.1016/j.abb.2013.07.002

[31] Vishnevetsky M , Ovadis M, Vainstein A. Carotenoid sequestration in plants: the role of carotenoid-associated proteins. Trends Plant Sci. 1999;4:232–5. 10366880 10.1016/s1360-1385(99)01414-4

[32] Li X , Sun J, Chen Z et al. Metabolite profile and genes/proteins expression in β-citraturin biosynthesis during fruit ripening in Chinese raspberry (*Rubus chingii* Hu). Plant Physiol Biochem. 2021;163:76–86. 33819717 10.1016/j.plaphy.2021.03.022

[33] Liang M-H , Xie H, Chen H-H et al. Functional identification of two types of carotene hydroxylases from the green alga Dunaliella bardawil rich in lutein. ACS Synth Biol. 2020;9:1246–53. 32408742 10.1021/acssynbio.0c00070

[34] Quinlan RF , Shumskaya M, Bradbury LMT et al. Synergistic interactions between carotene ring hydroxylases drive lutein formation in plant carotenoid biosynthesis. Plant Physiol. 2012;160:204–14. 22786888 10.1104/pp.112.198556 PMC3440199

[35] An J-P , Liu YJ, Zhang XW et al. Dynamic regulation of anthocyanin biosynthesis at different light intensities by the BT2-TCP46-MYB1 module in apple. J Exp Bot. 2020;71:3094–109. 31996900 10.1093/jxb/eraa056 PMC7475178

[36] Nuraini L , Ando Y, Kawai K et al. Anthocyanin regulatory and structural genes associated with violet flower color of *Matthiola incana*. Planta. 2020;251:1–15. 10.1007/s00425-020-03351-z 32036464

[37] Chen W , He S, Liu D et al. A sweetpotato geranylgeranyl pyrophosphate synthase gene, *IbGGPS*, increases carotenoid content and enhances osmotic stress tolerance in *Arabidopsis thaliana*. PLoS One. 2015;10:e0137623. 26376432 10.1371/journal.pone.0137623 PMC4574098

[38] Li R , Chen P, Zhu L et al. Characterization and function of the 1-deoxy-D-xylose-5-phosphate synthase (DXS) gene related to terpenoid synthesis in Pinus massoniana. Int J Mol Sci. 2021;22:848. 33467778 10.3390/ijms22020848 PMC7830437

[39] Cunningham FX Jr , Pogson B, Sun Z et al. Functional analysis of the beta and epsilon lycopene cyclase enzymes of Arabidopsis reveals a mechanism for control of cyclic carotenoid formation. Plant Cell. 1996;8:1613–26. 8837512 10.1105/tpc.8.9.1613 PMC161302

[40] Kang C , Zhai H, Xue L et al. A lycopene β-cyclase gene, *IbLCYB2*, enhances carotenoid contents and abiotic stress tolerance in transgenic sweetpotato. Plant Sci. 2018;272:243–54. 29807598 10.1016/j.plantsci.2018.05.005

[41] Zhang J , Tao N, Xu Q et al. Functional characterization of citrus PSY gene in Hongkong kumquat (*Fortunella hindsii* Swingle). Plant Cell Rep. 2009;28:1737–46. 19813015 10.1007/s00299-009-0774-3

[42] Zhu K , Zheng X, Ye J et al. Regulation of carotenoid and chlorophyll pools in hesperidia, anatomically unique fruits found only in citrus. Plant Physiol. 2021;187:829–45. 34608960 10.1093/plphys/kiab291 PMC8491056

[43] Tian H , Chen S, Yang W et al. A novel family of transcription factors conserved in angiosperms is required for ABA signalling. Plant Cell Environ. 2017;40:2958–71. 28857190 10.1111/pce.13058

[44] Yu B , Zhai H, Wang Y et al. Efficient agrobacterium tumefaciens-mediated transformation using embryogenic suspension cultures in sweetpotato, *Ipomoea batatas* (L.) *Lam*. Plant Cell Tissue Organ Cult. 2007;90:265–73.

[45] Zhang H , Gao X, Zhi Y et al. A non-tandem CCCH-type zinc-finger protein, IbC3H18, functions as a nuclear transcriptional activator and enhances abiotic stress tolerance in sweet potato. New Phytol. 2019;223:1918–36. 31091337 10.1111/nph.15925

[46] Zhang H , Zhang Q, Zhai H et al. IbBBX24 promotes the jasmonic acid pathway and enhances fusarium wilt resistance in sweet potato. Plant Cell. 2020;32:1102–23. 32034034 10.1105/tpc.19.00641 PMC7145486

[47] Schmittgen TD , Livak KJ. Analyzing real-time PCR data by the comparative CT method. Nat Protoc. 2008;3:1101–8. 18546601 10.1038/nprot.2008.73

[48] Li R , Kang C, Song X et al. A ζ-carotene desaturase gene, *IbZDS*, increases β-carotene and lutein contents and enhances salt tolerance in transgenic sweetpotato. Plant Sci. 2017;262:39–51. 28716419 10.1016/j.plantsci.2017.05.014

[49] Ma Y-N , Xu DB, Li L et al. Jasmonate promotes artemisinin biosynthesis by activating the TCP14-ORA complex in *Artemisia annua*. Sci Adv. 2018;4:eaas9357. 30627665 10.1126/sciadv.aas9357 PMC6317983

[50] Hellens RP , Allan AC, Friel EN et al. Transient expression vectors for functional genomics, quantification of promoter activity and RNA silencing in plants. Plant Methods. 2005;1:13. 16359558 10.1186/1746-4811-1-13 PMC1334188

