# Supplementary material for: The transcription factor IbNAC29 positively regulates the carotenoid accumulation in sweet potato
Source: Hortic Res. 2023 Feb 1;10(3):uhad010. doi: 10.1093/hr/uhad010 (PMC10028406; doi:10.1093/hr/uhad010)
Supplement: Web_Material_uhad010 [file web_material_uhad010.zip › Supplementary-0119.docx]

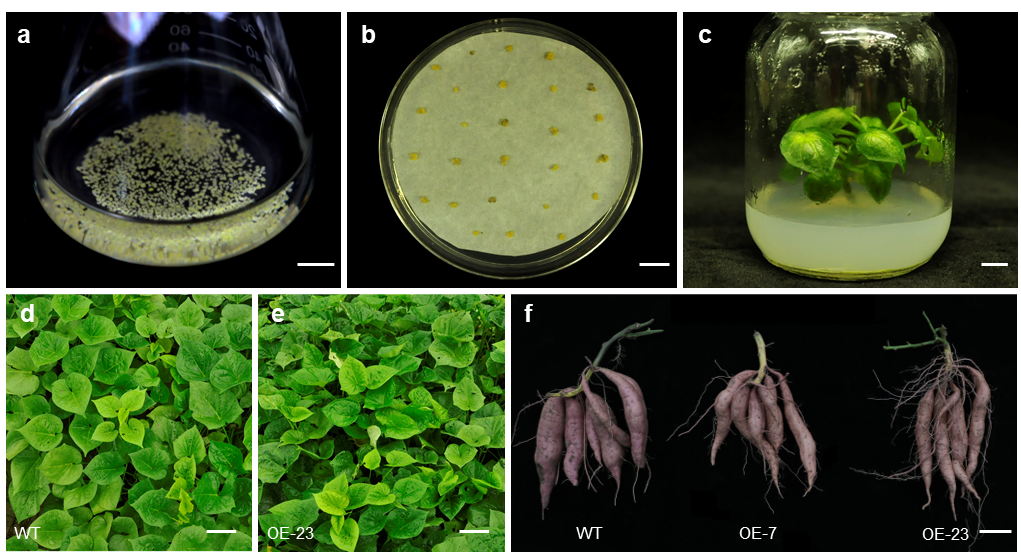


**Supplemental Figure S1** Generation of the *IbNAC29*-OE plants. **a** Embryogenic suspension cultures of sweet potato, Bar = 1 cm. **b** The formation of Hyg-resistant embryogenic calluses, Bar = 1 cm. **c** Plant regeneration, Bar = 1 cm.

**
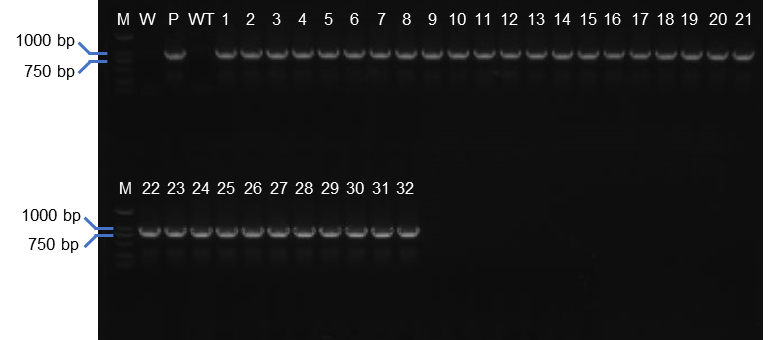
**

**Supplemental Figure S2** Identification of *IbNAC29*-OE plants by PCR. Lane M, DL 2000 bp marker. Lane W, Water (negative control). Lane P, Vectors pCAMBIA1300-IbNAC29 (positive control). Lane WT, Lizixiang (negative control). Lanes 1-32, independent *IbNAC29*-OE plants.

**
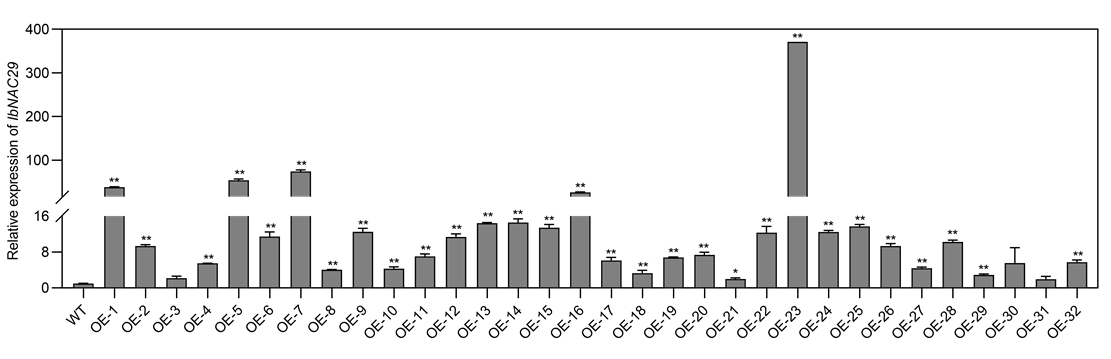
**

**Supplemental Figure S3** qRT-PCR analysis of *IbNAC29* mRNA levels in the leaves of the transgenic sweet potato plants. Error bars indicate SD (n = 3). * and ** indicate a significant difference from that of WT at *P* < 0.05 and *P* < 0.01, respectively, by Student’s *t*-test.


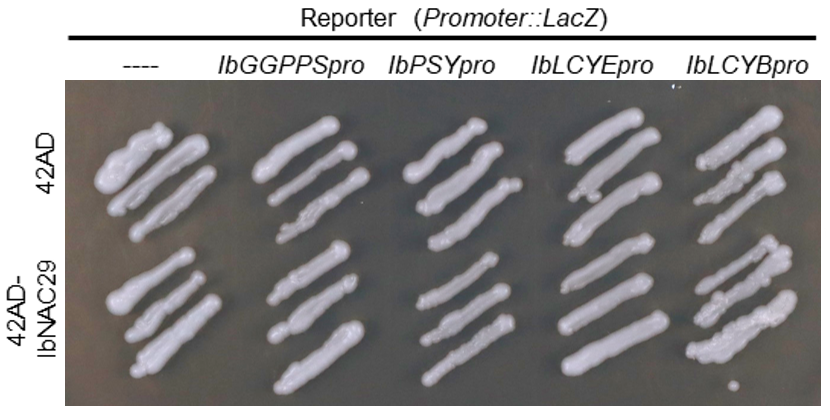


**Supplemental Figure S4** Y1H assay showed that 42AD-IbNAC29 did not activate the expression of the *LacZ* reporter genes driven by *IbGGPPS*, *IbPSY*, *IbLCYE,* and *IbLCYB* promoters in yeast cells.

**
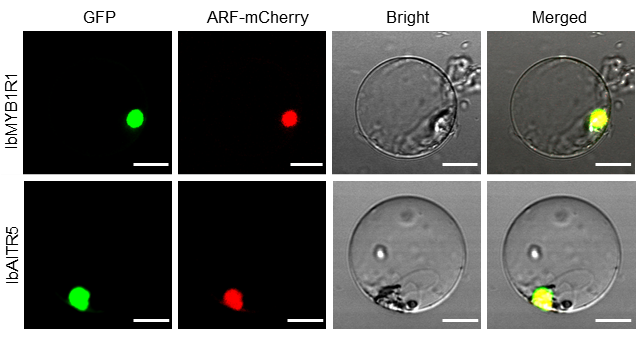
**

**Supplemental Figure S5** Subcellular localization of IbMYB1R1 and IbAITR5 in rice protoplasts. IbMYB1R1-GFP and IbAITR5-GFP were co-transformed with ARF-mCherry (a nuclear marker), respectively. Bar = 10 μm.


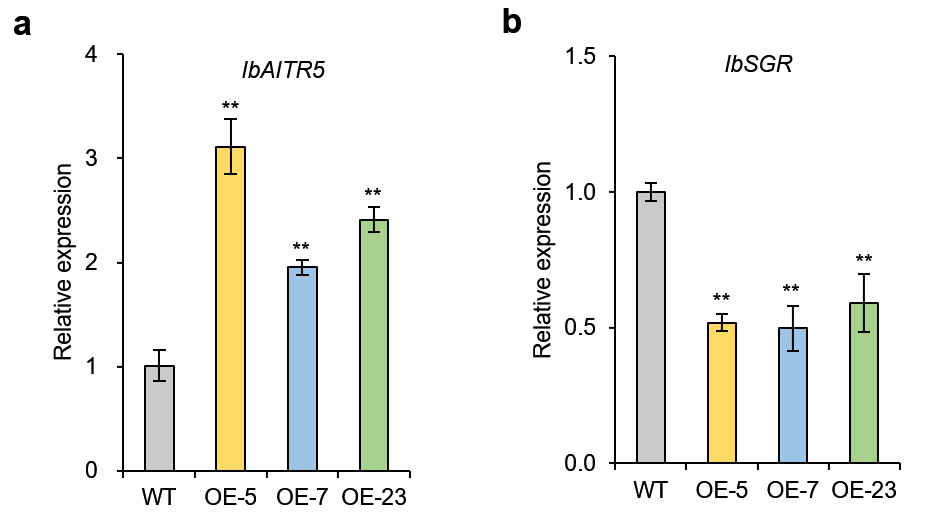


**Supplemental Figure S6** Transcript levels of *IbSGR1* (a) and *IbAITR5* (b) in *IbNAC29*-OE storage roots. Error bars indicate SD (n = 3). ** indicates *P* < 0.01, as determined by Student’s t-test analysis.

**Supplemental Table S1 Primers used in this study**

| Primer name | Primer sequence (5’-3’) |
| --- | --- |
| Primers for 5’/3’ RACE | |
| *IbNAC29*-GSP5-1 | GGGCTGGTTGAAGATGCT |
| *IbNAC29*-GSP5-2 | TTGCCTGGCGGGTAATGC |
| *IbNAC29*-GSP3-1 | AATAGGGCAGCCGTGTCG |
| *IbNAC29*-GSP3-2 | TAGTCCCACATCAATACAGC |
| Primers for constructing vectors | |
| *IbNAC29*-CDS-F | ATGGTGGGCGGCAAACAAA |
| *IbNAC29*-CDS-R | TCACTGGAATTGGAAAGCTGG |
| *IbMYB1R1-*CDS-F | ATGGGAAGAAAATGCTCACATTGT |
| *IbMYB1R1-*CDS*-*R | CTACACATTATTCTTGGGAGCTCC |
| *IbAITR5*-CDS-F | ATGGATGGGCGAGGGGG |
| *IbAITR5*-CDS-R | CTATAAGGATAGAGCAGCCTCTGTA |
| *IbSGR1-*CDS*-*F | ATGGGGACTTTATCTGCCTCTC |
| *IbSGR1-*CDS*-*R | TTAGTTTAGTTTAGCCTTAGCCACC |
| *IbNAC29*-GD-F | CGGAAACACCTCATTTTCCTT |
| *IbNAC29*-GD-R | CGCGGATCCTCCACTAGTGA |
| pCAMBIA1300-IbNAC29-F-*Kpn* I | GGGGTACCATGGTGGGCGGCAAACAAA |
| pCAMBIA1300-IbNAC29-R-*Bam*H I | CGGGATCCCTGGAATTGGAAAGCTGG |
| pCAMBIA1300-IbMYB1R1-F-*Kpn* I | ACGGGGGACGAGCTCGGTACCATGGGAAGAAAATGCTCACATTGT |
| pCAMBIA1300-IbMYB1R1-R-*Sal* I | GCCCTTGCTCACCATGTCGACCACATTATTCTTGGGAGCTCCA |
| pCAMBIA1300-IbAITR5-F-*Kpn* I | ACGGGGGACGAGCTCGGTACCATGGATGGGCGAGGGGG |
| pCAMBIA1300-IbAITR5-R-*Sal* I | GCCCTTGCTCACCATGTCGACTAAGGATAGAGCAGCCTCTGTA |
| pGBKT7-IbNAC29-F-*Nde* I | GGAATTCCATATGATGGTGGGCGGCAAACAAA |
| pGBKT7-IbNAC29-R-*Bam*H I | CGGGATCCTCACTGGAATTGGAAAGCTGG |
| pGBKT7-IbAITR5-F-*Eco*R I | GGAATTCATGGATGGGCGAGGGGG |
| pGBKT7-IbAITR5-R-*Pst* I | AACTGCAGCTATAAGGATAGAGCAGCCTCTGTA |
| pGAD-IbMYB1R1-F- *Eco*R I | GCCATGGAGGCCAGTGAATTCATGGGAAGAAAATGCTCACATT |
| pGAD-IbMYB1R1-R- *Bam*H I | CAGCTCGAGCTCGATGGATCCCTACACATTATTCTTGGGAGCTCC |
| pGAD-IbAITR5-F- *Eco*R I | GCCATGGAGGCCAGTGAATTCATGGATGGGCGAGGGG |
| pGAD-IbAITR5-R- *Bam*H I | CAGCTCGAGCTCGATGGATCCCTATAAGGATAGAGCAGCCTCTGTA |
| pBridge-IbNAC29-F-*Eco*R I | GGTCAAAGACAGTTGACTGTATCGCCGGAATTCATGGTGGGCGGCAAACAAA |
| pBridge-IbNAC29-R-*Bam*H I | GAATTAGCTTGGCTGCAGGTCGACGGATCCTCACTGGAATTGGAAAGCTGG |
| pBridge-IbNAC29-IbMYB1R1-F-*Not* I | CTTCTAGTCCTAAGAAGAAGAGAAAGGTGGCGGCCGCATGGGAAGAAAATGCTCACATTGT |
| pBridge-IbNAC29-IbMYB1R1-R-*Bgl* II | AGTAGAGACATGGGAGATCAGCCCGAAGATCTCTACACATTATTCTTGGGAGCTCCA |
| pSPYNE-35S-IbNAC29-F-*Asc* I | ACCGGGCTCAGGCCTGGCGCGCCATGGTGGGCGGCAAACAAA |
| pSPYNE-35S-IbNAC29-R-*Kpn* I | CTCCATCCCGGGAGCGGTACCCTGGAATTGGAAAGCTGG |
| pSPYNE-35S-IbAITR5-F-*Asc* I | ACCGGGCTCAGGCCTGGCGCGCCATGGATGGGCGAGGGG |
| pSPYNE-35S-IbAITR5-R-*Kpn* I | CTCCATCCCGGGAGCGGTACCTAAGGATAGAGCAGCCTCTGTA |
| pSPYCE-35S-IbMYB1R1-F-*Asc* I | ACCGGGCTCAGGCCTGGCGCGCCATGGGAAGAAAATGCTCACATTGT |
| pSPYCE-35S-IbMYB1R1-R-*Kpn* I | GTACATCCCGGGAGCGGTACCCACATTATTCTTGGGAGCTCCA |
| IbNAC29-HA-F-*Spe* I | GACGAGCTCTCTAGAACTAGTATGGTGGGCGGCAAACAAA |
| IbNAC29-HA-R-*Kpn* I | TTTGCGGAGTACCCGGGTACCTCACTGGAATTGGAAAGCTGGG |
| IbAITR5-HA-F-*Spe* I | GACGAGCTCTCTAGAACTAGTATGGATGGGCGAGGGG |
| IbAITR5-HA-R-*Kpn* I | TTTGCGGAGTACCCGGGTACCCTATAAGGATAGAGCAGCCTCTGTA |
| IbMYB1R1-MYC-F-*Hin*d III | CAAATCGACTCTAGAAAGCTTATGGGAAGAAAATGCTCACATTGT |
| IbMYB1R1-MYC-R-*Kpn* I | GAGCTTTTGCTCCATGGTACCCACATTATTCTTGGGAGCTCCA |
| pB42AD-IbNAC29-F-*Eco*R I | GATTATGCCTCTCCCGAATTCATGGTGGGCGGCAAACAAA |
| pB42AD-IbNAC29-R-*Xho* I | AGAAGTCCAAAGCTTCTCGAGCTGGAATTGGAAAGCTGG |
| pB42AD-IbMYB1R1-F-*Eco*R I | GATTATGCCTCTCCCGAATTCATGGGAAGAAAATGCTCACATTGT |
| pB42AD-IbMYB1R1-R-*Xho* I | AGAAGTCCAAAGCTTCTCGAGCACATTATTCTTGGGAGCTCCA |
| pB42AD-IbAITR5-F-*Eco*R I | GATTATGCCTCTCCCGAATTCATGGATGGGCGAGGGG |
| pB42AD-IbAITR5-R-*Xho* I | AGAAGTCCAAAGCTTCTCGAGTAAGGATAGAGCAGCCTCTGTA |
| pLacZi2μ-IbGGPPS-F-*Eco*R I | TTTGATATTGGATCGGAATTCAAGGAATGTCGGCCATCAAAGGAGC |
| pLacZi2μ-IbGGPPS-R-*Xho* I | ATACAGAGCACATGCCTCGAGTTTCTGATTCTGAAGAATTCCAA |
| pLacZi2μ-IbPSY-F-*Eco*R I | TTTGATATTGGATCGGAATTCTTTTAGTTACTGGTCGCCGTGCCG |
| pLacZi2μ-IbPSY-R-*Xho* I | ATACAGAGCACATGCCTCGAGACTTGAGCTGAGGTGAAATAG |
| pLacZi2μ-IbLCYE-F-*Eco*R I | TTTGATATTGGATCGGAATTCTTAAACCTAAAAGCAACGAATA |
| pLacZi2μ-IbLCYE-R-*Xho* I | ATACAGAGCACATGCCTCGAGTTCTGCCCGGAATCCTCGCTTT |
| pLacZi2μ-IbLCYB-F-*Eco*R I | TTTGATATTGGATCGGAATTCTATGTATATTTAGTTATAAG |
| pLacZi2μ-IbLCYB-R-*Xho* I | ATACAGAGCACATGCCTCGAGCACCCTTCCACACTACAATCTG |
| pLacZi2μ-IbSGR1-F-*Eco*R I | TTTGATATTGGATCGGAATTCGCTACGTGGAGAAGGGTAAACAGTG |
| pLacZi2μ-IbSGR1-R-*Xho* I | ATACAGAGCACATGCCTCGAGCCAGAGAGGCAGATAAAGTCCCCAT |
| pBD-GAL4-IbNAC29-F-*Eco*R I | TCCCCCGGGCTGCAGGAATTCATGGTGGGCGGCAAACAAA |
| pBD-GAL4-IbNAC29-R-*Kpn* I | TTTGCGGAGTACCCGGGTACCCTGGAATTGGAAAGCTGG |
| pGreenII-62SK-IbNAC29-F-*Pst* I | GTGGATCCCCCGGGCTGCAGATGGTGGGCGGCAAACAAA |
| pGreenII-62SK-IbNAC29-R-*Kpn* I | GATTTCAGCGAATTGGTACCTCACTGGAATTGGAAAGCTGG |
| pGreenII-62SK-IbMYB1R1-F-*Pst* I | GTGGATCCCCCGGGCTGCAGATGGGAAGAAAATGCTCACATTGT |
| pGreenII-62SK-IbMYB1R1-R-*Kpn* I | GATTTCAGCGAATTGGTACCCTACACATTATTCTTGGGAGCTCCA |
| pGreenII-62SK-IbAITR5-F-*Pst* I | GTGGATCCCCCGGGCTGCAGATGGATGGGCGAGGGG |
| pGreenII-62SK-IbAITR5-R-*Kpn* I | GATTTCAGCGAATTGGTACCCTATAAGGATAGAGCAGCCTCTGTA |
| pGreenII-62SK-IbSGR1-F-*Pst* I | GTGGATCCCCCGGGCTGCAGATGGGGACTTTATCTGCCTCTC |
| pGreenII-62SK-IbSGR1-R-*Kpn* I | GATTTCAGCGAATTGGTACCTTAGTTTAGTTTAGCCTTAGCCACC |
| pGreenII-0800-IbSGR1-F-*Kpn* I | TATAGGGCGAATTGGGTACCAGGTCGTGAATACTAACTTCCATCT |
| pGreenII-0800-IbSGR1-R-*Pst* I | GTGGATCCCCCGGGCTGCAGTGTTATAAGCTCAATTACCCTTCAA |
| pGreenII-0800-IbPSY-F-*Kpn* I | TATAGGGCGAATTGGGTACCCACTGCGAGTCGAACTCAC |
| pGreenII-0800-IbPSY-R-*Pst* I | GTGGATCCCCCGGGCTGCAGTCTTGAGGTGAAATAGTTTCTATAT |
| pGEX-6P-1-IbAITR5-F-*Eco*R I | GGAATTCATGGATGGGCGAGGGGG |
| pGEX-6P-1-IbAITR5-R-*Xho* I | CCGCTCGAGCTATAAGGATAGAGCAGCCTCTGTA |
| Primers for identifying transformants | |
| 35S-F | GACGCACAATCCCACTATCC |
| *IbNAC29*-R | TCACTGGAATTGGAAAGCTGG |
| Primers for qRT-PCR | |
| *IbActin*-F | AGCAGCATGAAGATTAAGGTTGTAGCAC |
| *IbActin*-R | TGGAAAATTAGAAGCACTTCCTGTGAAC |
| *IbNAC29*-F | AAGTTGTTCATCAGAGGGTGG |
| *IbNAC29*-R | GGTTGTCAAAGGTTGCGTTA |
| *IbDXS-*F | CCACAAACCTGCTCAAAT |
| *IbDXS*-R | GCCACCACCTAAATCAAA |
| *IbGGPPS-*F | TGTTAATTCTTGGGCCCGGA |
| *IbGGPPS-*R | TCCACACTAACCCTCACACC |
| *IbPSY*-F | GGCAGGGCTAAGAAGGGTAA |
| *IbPSY*-R | GATGACATCGCCATTTCCCC |
| *IbLCYE*-F | CGCAAGAAAGGAAACGACAA |
| *IbLCYE*-R | GCGGAAGAATGTGTGGAAAA |
| *IbCYP97A3*-F | CTTGCCATTTGGTGGAGG |
| *IbCYP97A3*-R | GAATTGTTGCCCCTGTCG |
| *IbCYP97C1*-F | AGCTTGAGGACGTGTCGG |
| *IbCYP97C1*-R | GCAACAAGCCCCTTAGCA |
| *IbLCYB*-F | GACGAGCGTTGTGTTATCCC |
| *IbLCYB*-R | CTCCTCTCTATCGGCCACAG |
| *IbBCH*-F | CTGAGCTCTCGGAGAAAACCT |
| *IbBCH*-R | GATCTTTTCCTGGCCAATTTC |
| *IbZEP*-F | TGGTACTTGGATCACCGACA |
| *IbZEP*-R | GCTGCCTGCAAAACTTTCAT |
| *IbCCS*-F | TAGCCGAGGCCATAGCTG |
| *IbCCS*-F | TGAGGAGAGGAAGCCGTG |
| *IbSGR1*-F | TGCCTCTCTGGTGCTTCC |
| *IbSGR1-*R | TGGAAGCTTTCCTGGGTG |
| *IbAITR5*-F | CGGATGGATCTGTTTCCG |
| *IbAITR5-*R | ACTTGACACCGACGAGCC |
| Primers for ChIP-qRT | |
| P1-F | GCTGATAGTGTTAATTCCTTTGGTG |
| P1-R | CTCCTAAACTCCCTCCAAAAGATTC |
| P2-F | ACTAACTTCCATCTCTACCCCTTGT |
| P2-R | TTGTTATAAGCTCAATTACCCTTCA |
